# Inclusive climates and employee voice behavior: the roles of voice efficacy and voice channel design

**DOI:** 10.3389/fpsyg.2025.1681910

**Published:** 2025-12-09

**Authors:** Weiyi Shen, Lidan Shen

**Affiliations:** School of International and Public Affairs, Shanghai Jiao Tong University, Shanghai, China

**Keywords:** inclusive climates, voice behavior, voice efficacy, voice channel design, university employee

## Abstract

**Introduction:**

Employee voice serves as a critical source of organizational competitiveness. Scholars have discussed how to enhance employees' willingness to engage in voice behavior from multiple perspectives. But few studies have investigated the impact of voice channel design practices on employees' voice behavior. Based on sense-making theory, this study examined the effect of the organizational climate of inclusivity on voice behavior, with voice efficacy as a mediator and satisfaction with the design of voice channels as a moderator.

**Methods:**

Using data collected from a sample of 281 university employees in eastern China, we analyzed a moderated mediation model.

**Results:**

The empirical results showed a significant relationship between an inclusive work climate and voice behavior. Voice efficacy played a partial mediation role in the relationship between an inclusive climate and voice behavior. Satisfaction with the design of voice channels positively moderated the positive effect of a climate for inclusion on voice behavior through voice efficacy. The moderating effect of satisfaction with a formal voice channel was more significant than that of satisfaction with an informal voice channel.

**Discussion:**

The findings in this study may inform ways of motivating employees to proactively voice their opinions to promote the development of their organization. By fostering an inclusive organizational climate and designing effective voice mechanisms, organizations can strengthen employees' confidence in their capacity to make meaningful contributions, thereby elucidating how prior voice experiences influence subsequent voice-related decisions.

## 1 Introduction

Given the challenges of the dynamic environments in which organizations operate, organizations need the support of employees who have the capability, and are willing, to take on a broader role. Employees are expected to display proactive behaviors and take the initiative in their work (Parker, 1998). Voicers can proactively initiate dialogue by proposing ideas designed to functionally enhance the work environment (Kim et al., 2023; Rubenstein et al., 2025). As a type of challenging promotive behavior (Van Dyne and LePine, 1998), voice behavior has been defined as informal and discretionary communication of ideas, suggestions, concerns, problems, or opinions about work-related issues, with the intention of facilitating improvement or change (Morrison, 2023).

According to sense-making theory, people interpret clusters of related events to form context-based schemas that guide their future decisions and behaviors at work (Schneider and Reichers, 1983). Individuals give meaning to past events and explain the environment through constructing meaning (Weick, 1995). The sense makers interpret the world (Baillie and Corrie, 1996) through the lens of, or the schemata derived from, their prior experience. The interpretation and understanding of the environment is then rationalized to affect the cognition, attitude, and behavior of individuals around the events they are experiencing.

The climate of the work environment is defined and shaped by the individuals within it (Xu et al., 2019). Management practices designed to create an inclusive climate can play a significant role in facilitating the positive outcomes associated with diversity, including improved job satisfaction, heightened creativity, and increased employee retention, while simultaneously reducing negative consequences such as distrust and communication inefficiencies (Mor Barak et al., 2016). Nishii (2013) defined a climate for inclusion as a type of focused climate where organizational members feel treated with equity and impartiality, recognized for having a unique identity, and integrated into the decision making. It has been suggested that an inclusive climate should be maintained to enable employees to express their opinions in a psychologically safe environment, as individuals who perceive higher levels of psychological safety are more likely to engage in voice behaviors in the workplace (Edmondson and Lei, 2014; Ge, 2020).

Organizational practices can promote desired proactive behaviors by participating in “sense-giving” at the individual level (Maitlis, 2005). Individuals engage in a sense-making process by interpreting personal information and situational cues, and use these interpretations to form efficacy judgments (Maitlis and Christianson, 2014). On the one hand, voice channel design is a human resources practice that can facilitate employee voice behavior (Mowbray et al., 2021). Satisfaction with the design of voice channels reflects how employees make sense of contextual arrangements such as specific “channels of voice” (Pyman et al., 2006) or “voice pathways” (Townsend et al., 2022) based on their personal experiences. On the other hand, inclusive organizational practices empower employees to perceive their competence in engaging in the organization's decision-making processes, a perception referred to as voice efficacy, and thereby strengthen their confidence that their actions and initiatives will yield meaningful and effective outcomes (Shore et al., 2011).

Therefore, individuals in diverse workplace settings will make sense of previous voice experiences by identifying satisfaction with voice channel design within the organizational context in order to enhance voice efficacy to shape subsequent voice behaviors. As discussed above, this study aims to build a moderated mediation model positioning voice efficacy as a mediator of the climate for inclusion's effects on voice behavior, and satisfaction with voice channel design as a moderator of such effect.

## 2 Literature review and hypotheses development

### 2.1 Climate for inclusion and voice behavior

Human resource development plays a critical role in cultivating intelligent and highly skilled employees within organizations. To facilitate employees to effectively contribute to the organization's goal attainment, it is essential for the organization to create an environment conducive to fostering proactive behaviors (Mubarak et al., 2021). There is growing evidence that employees are more likely to engage in proactive behavior in an environment with a higher level of inclusivity. From the perspective of inclusive leadership, the quality of the leader-member exchange significantly influences individual employee behaviors or organizational-level outcomes (Mustafa et al., 2023). Previous studies have demonstrated that inclusive leadership effectively promotes employees' proactive behaviors (e.g., innovative work behavior) (Mir et al., 2021; Khan et al., 2024). Empirical evidence also showed that inclusive leadership is positively associated with organizational success (e.g., project success) (Khan et al., 2020). From the perspective of inclusive organizational climate, inclusion refers to the creation of an inclusive organizational culture in which diverse individuals and groups are able to work effectively and thrive (Pelled et al., 1999; Roberson, 2006). A climate for inclusion refers to the extent to which an employee perceives their acceptance by colleagues as an insider within the workplace (Hope Pelled et al., 1999). There are three dimensions to a climate for inclusion: equitable employment practices, integration of differences, and inclusion in decision-making (Le et al., 2021). Since employees should have a voice in the broader operations of their organizations (Wood and Wall, 2007), a climate for inclusion should be cultivated within the context of diversity management to enable every member of the workforce to perform to their full potential (Olsen and Martins, 2012). We therefore proposed the following hypothesis:

***Hypothesis 1***: A climate for inclusion will positively influence voice behavior.

### 2.2 The mediating role of voice efficacy

Self-efficacy is the judgments of individual's ability to perform specific tasks, including assessments of skills, adaptability, creativity, and the capacity to maintain self-control under stress (Locke and Bandura, 1987). Perceived self-efficacy and personal goal-setting enhance motivation and behavior, leading to improved performance (Bandura and Locke, 2003). Voice efficacy can be defined as a belief in one's competence to speak up effectively and to achieve positive outcomes (Kish-Gephart et al., 2009). Organizational factors such as a shared belief that speaking up is encouraged (group voice climate) are predictors of voice behaviors (Morrison et al., 2011). We therefore proposed the following hypothesis:

***Hypothesis 2***: A climate for inclusion will positively influence voice efficacy.

Voice behavior is a form of extra-role behavior that constructively challenges the status quo, This requires strong motivation and the capacity to express suggestions, and can therefore be predicted by psychological antecedents (Liang et al., 2012). Employee self-efficacy is expected to be positively associated with employee voice behavior (Du and Bao, 2023). Employees with a high level of voice efficacy perceive themselves as capable and knowledgeable enough to propose constructive ideas at work and believe that their opinions will be taken seriously (Tangirala et al., 2013). We therefore proposed the following hypothesis:

***Hypothesis 3***: Voice efficacy will positively influence voice behavior.

Voice efficacy demonstrates an individual's judgments about whether they have the ability to perform the voice behavior. Research has revealed that individual voice efficacy is largely shaped by contextual constraints (Milliken et al., 2003), and is closely associated with specific contextual characteristics, such as voice systems and structures (Huang et al., 2024). In other terms, the organization's culture is one of the motivational factors that may enhance an employee's ability to speak up in the workplace (Rani et al., 2024). Employees with high levels of self-esteem or work-related efficacy are more inclined to effectively articulate their ideas and suggestions in different cultural contexts (Detert and Burris, 2007; Duan et al., 2014). Based on this, it can be inferred that self-efficacy serves as a critical mediating mechanism explaining how contextual factors drive employee voice (Zhang et al., 2019).

Studies have shown that voice is more prevalent in the context of managerial practices designed to enhance employee skills, motivation, or involvement (Chamberlin et al., 2018). Organizations that adhere to best practices for cultivating a highly engaged workforce are likely to create environments that encourage greater voice and less silence (Morrison, 2023). Although few researchers have directly examined how employees are motivated to speak up in an inclusive climate, it has been demonstrated that the delegation of responsibility, authority, and decision-making power positively influences an individual's propensity to engage in voice behavior through the mediating effect of the felt obligation for constructive change and the moderating effect of the voice climate (Rubbab et al., 2023). There is also evidence that an organization with a culture of employee autonomy can enhance employees' self-efficacy, reduce their psychological stress, and thereby encourage them to speak up (Dedahanov et al., 2019). We therefore proposed the following hypothesis:

***Hypothesis 4:*** Voice efficacy plays a mediating role between a climate for inclusion and voice behavior.

### 2.3 The moderating role of satisfaction with voice channel design

The initiative in offering suggestions is not only demonstrated by individuals' communicating their perspectives on specific work issues, but also by their deliberate selection of particular channels to articulate and convey their viewpoints and opinions. Voice channels are a means of improving the chances of their voice being heard and their concerns being addressed (Mowbray, 2018). The organizational norms associated with different voice channels convey signals to employees regarding the consequences of voicing their opinions, specifically the safety and effectiveness of doing so (Kwon and Farndale, 2020). The repeated successful, or even partially successful, experiences of speaking up can develop individuals' voice efficacy (Kish-Gephart et al., 2009).

An inclusive climate, facilitated by workplace information exchange channels, reduces the perception of individual differences among group members and promotes equal and free participation in discussions and decision-making (Gallegos, 2014). This would suggest that creating a range of channels would enable employees to express their opinions, fostering positive emotional experiences and enhancing their sense of competence during the voice process. We therefore proposed the following hypothesis:

***Hypothesis 5***: Satisfaction with voice channel design positively moderates the relationship between an inclusive climate and voice efficacy. The higher the satisfaction with the design of the voice channels, the stronger the relationship between the climate for inclusion and voice efficacy.

Since inclusion refers to the individual's perception of being a part of both the formal and informal processes of an organizational system (Mor Barak and Cherin, 1998), it could be suggested that the climate for inclusion should enable individuals to express themselves through formal and informal channels (Zhang et al., 2019). Formal mechanisms that enable employee input may promote upward communication and strengthen employee involvement in strategic issues. Informal mechanisms may empower employees to perceive opportunities for their voices to be heard and to influence the functioning and success of organizations (Roberson and Scott, 2024). Formal and informal voice channels are two types of voice mechanisms composed of multiple organizational practices that encourage employees to speak up (Huang et al., 2024).

Salancik and Pfeffer (1978) stated that attitudes and behavior at work were shaped by how employees processed and interpreted the information available in the social environment of the workplace. King et al. (2019) suggested that the adoption of suggestions employees had previously proposed would prompt individuals to reflect on their prior voice experience. Accumulated prior experiences (direct and indirect) of a particular situation or stimulus could develop into an automatic mood association based on cues from those past experiences. The long-term memory of voice experiences via specific voice channels could prompt individuals to form attitudes toward subsequent voice behavior and its possible outcomes. This would point to the role of voice channels in enhancing voice efficacy in an organization.

Based on the above analysis, voice efficacy may play a mediating role between the perception of an inclusive climate and voice behavior. Satisfaction with voice channel design may moderate the relationship between the climate for inclusion and voice efficacy. Our study, therefore, constructed a moderated mediation model.

When the level of actual employee voicing in the organization is high, the proactive motivation of voice behavior can be cultivated over time and thus demonstrates self-amplifying patterns (Li and Tangirala, 2021). When the voice channel helps individuals develop positive experiences of speaking up, these channels may serve as a reinforcement mechanism for voice behavior. Therefore, satisfaction with voice channel design may facilitate individuals to transform their voice efficacy into voice behaviors. We therefore proposed the following hypothesis:

***Hypothesis 6***: Satisfaction with voice channel design positively moderates the mediating effect of voice efficacy between the climate for inclusion and voice behavior. The higher the satisfaction with the voice channel design, the stronger the mediating effect of voice efficacy. The lower the satisfaction, the weaker the mediating effect of voice efficacy.

**Figure 1:**
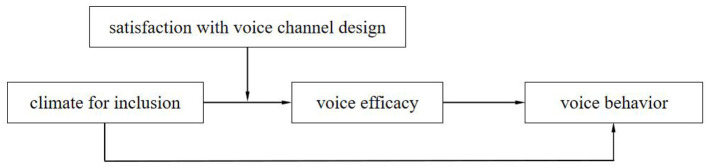
Theoretical model.

The theoretical model is shown in Figure 1.

## 3 Method

### 3.1 Participants and procedure

Due to limitations in resources and time, we selected the university faculty and staff as the sample source for this study. We used convenience sampling for this study, inviting employees from one of the high-level universities in eastern China to complete an online questionnaire which took one and a half months. This university ranks among the oldest in China and is widely acknowledged as a leading institution in the national higher education system, reflecting its enduring academic excellence and historical significance. The selected university has implemented institutional reforms aimed at optimizing its internal governance structure, thereby stimulating the innovative capacity of grassroots academic units, enhancing the initiative of faculty and staff, and has developed practical experiences for other universities to learn from.

Before beginning the formal survey, we sought and received ethical approval from Institutional Review Board for Human Research Protections of our university. Respondents participated voluntarily and were assured of the confidentiality of their personal information. To mitigate potential biases and limitations arising from the use of convenience sampling, we collected data from different groups. After rigorous screening and exclusion of invalid questionnaires for abnormal completion times and incorrect answers to reverse questions, there were 281 valid forms. The final sample comprised 157 women (55.9%) and 124 men (44.1%) with a mean age of 38.40 years (SD = 6.92, range = 25–65). The mean length of time in work was 7.67 years (SD = 6.33, range = 0–30, 0 means less than 1 year); 64.4% held doctoral degrees, 33.1% held master's degrees, and 2.5% held bachelor's degrees. Faculties constituted 64.8% (*n* = 182), and administrative staff represented 35.2% (*n* = 99). Furthermore, 32.4% of participants reported prior or current experience in management positions (*n* = 91), whereas 67.6% did not (*n* = 190).

### 3.2 Measures

We adopted scales that previous studies had shown to have high levels of reliability and validity. Some statements from these were slightly modified to fit our research context. We employed standard translation and back-translation procedures, along with expert recommendations, to translate the items from English into Chinese, ensuring linguistic accuracy and conceptual equivalence (Klotz et al., 2023). Items were evaluated on a 6-point Likert scale ranging from 1 (strongly disagree) to 6 (strongly agree). To reduce social desirability bias, this study clearly indicated at the beginning of the questionnaire that the collected data would be used exclusively for academic research purposes and could not be traced back to individuals.

#### 3.2.1 Climate for inclusion

We measured climate for inclusion with the scale proposed by Nishii (2013). An example item was: “This unit provides safe ways for employees to voice their grievances.” The Cronbach's alpha for this scale was 0.942.

#### 3.2.2 Voice efficacy

By integrating the general self-efficacy scale (Schwarzer et al., 1997) and voice behavior scale (Van Dyne and LePine, 1998) and drawing on related studies conducted in Chinese organizations, we assessed voice efficacy with six items. An example item was: “I am capable of seizing all appropriate opportunities to express my thoughts to the unit.” The Cronbach's alpha for this scale was 0.947.

#### 3.2.3 Satisfaction with voice channel design

The study was conducted at a public university in China, which is generally categorized as a public institution. Public institutions, unlike those in the private sector, feature more complex and hierarchical organizational structures, as well as more diversified channels for opinion expression. Nonetheless, organizational members may still remain silent due to specific organizational cultures and structural designs. Evidently, investigating whether the design of diverse types of voice channels can contribute to boosting the proactivity of organizational members in voicing their suggestions holds significant practical implications for the public sector.

To measure satisfaction with voice channel design, we used the classification of formal and informal channels from a previous study in which formal channels were defined as formal structures and mechanisms, and informal channels were defined as informal interactions between managers and staff at work (Mowbray et al., 2015). In this study, formal voice channels referred to mechanisms for expressing opinions through the Teachers and Staff Representative or General Assembly, while informal voice channels referred to mechanisms for conveying views through routine interpersonal interactions. Appropriate to management practices in this Chinese context, we used six items to evaluate satisfaction with formal voice channel design (e.g., “I am satisfied with the voice experience by approving and adopting the important reform plans of this unit at Teachers and Staff Representative or General Assembly”) and five items to evaluate satisfaction with informal channel design (e.g., “I am satisfied with the voice experience by communicating with the leaders of this unit”). The Cronbach's alpha for the scale of satisfaction with formal voice channel design was 0.918 and the Cronbach's alpha for the scale of satisfaction with informal voice channel design was 0.920.

#### 3.2.4 Voice behavior

We drew on the scale developed by Van Dyne and LePine (1998) and relevant studies in the context of China to measure voice behavior. A sample item for voice behavior was “I can develop and make recommendations concerning issues that affect this work group.” The Cronbach's alpha for this scale was 0.908.

## 4 Results

### 4.1 Reliability and validity testing

The reliability, convergent validity, and discriminant validity of the constructed measurement model were tested using SPSS and Mplus software. Reliability was evaluated by calculating Cronbach's alpha and composite reliability, convergent validity was assessed via the average variance extracted (AVE), and discriminant validity was examined using the Fornell–Larcker criterion. The results are shown in Table 1. Both Cronbach's alpha and composite reliability values for the five main constructs exceeded the critical threshold of 0.70, indicating acceptable levels of goodness of fit (Hair et al., 2013). For convergent validity, we found that the AVE values for all variables exceeded the threshold of 0.50, suggesting a satisfactory level of convergent validity (Henseler et al., 2009).

**Table 1:** Reliability and validity of the scales.

**Variable**	**Cronbach's alpha**	**CR**	**AVE**
IC	0.942	0.944	0.630
IFC	0.920	0.921	0.700
FC	0.918	0.922	0.665
VE	0.947	0.948	0.753
VC	0.908	0.910	0.627

CR, composite reliability; AVE, average variance extracted; IC, climate for inclusion; IFC, satisfaction with informal voice channel design; FC, satisfaction with formal voice channel design; VE, voice efficacy; VC, voice behavior.

We confirmed adequate discriminant validity among the constructs given that the square roots of the AVE values for all constructs in the model were greater than the correlations with other constructs (Fornell and Larcker, 1981). The correlation coefficients and descriptive statistics are presented in Table 2.

**Table 2:** Descriptive statistics and correlation matrix.

**Variable**	**M**	**SD**	**1**	**2**	**3**	**4**	**5**	**6**	**7**	**8**	**9**
1. Gender	1.56	0.50	1								
2. Age	38.40	6.92	−0.043	1							
3. Level of education	2.62	0.54	−0.244^**^	0.137^*^	1						
4. Working years	7.67	6.33	0.080	0.689^**^	−0.149^*^	1					
5. IC	4.48	0.89	−0.075	0.093	−0.122^*^	0.053	(0.794)				
6. IFC	4.64	0.95	−0.035	0.000	−0.128^*^	−0.018	0.700^**^	(0.837)			
7. FC	4.76	0.78	0.040	0.017	−0.169^**^	0.024	0.703^**^	0.561^**^	(0.815)		
8.VE	4.05	1.10	−0.112	0.109	−0.137^*^	0.068	0.710^**^	0.621^**^	0.500^**^	(0.868)	
9. VC	4.96	0.66	−0.060	−0.034	−0.173^**^	0.029	0.549^**^	0.392^**^	0.422^**^	0.566^**^	(0.792)

The square roots of the average variance extracted are given within the brackets on the diagonal. IC, climate for inclusion; IFC, satisfaction with informal voice channel design; FC, satisfaction with formal voice channel design; VE, voice efficacy; VC, voice behavior.

^**^p < 0.01.

^*^p < 0.05.

We also conducted a confirmatory factor analysis to test discriminant validity (see Table 3). In the measurement model in which satisfaction with informal voice channel design was regarded as the moderating variable, it was empirically demonstrated that the four-factor model (climate for inclusion, satisfaction with informal voice channel design, voice efficacy, voice behavior) exhibited a better fit to the actual data compared to alternative models, minimum discrepancy (CMIN) = 785.546, *df* = 318, CMIN/*df* = 2.47, Tucker-Lewis index (TLI) = 0.921, comparative fit index (CFI) = 0.928, root-mean-square error of approximation (RMSEA) = 0.072, indicating good discriminant validity. Meanwhile, in the measurement model that satisfaction with formal voice channel design is specified as the moderating variable, the four-factor model (climate for inclusion, satisfaction with formal voice channel design, voice efficacy, voice behavior) demonstrated the optimal level of fit among the candidate factor models and significantly outperformed the alternatives, minimum discrepancy (CMIN) = 828.054, *df* = 318, CMIN/*df* = 2.60, Tucker-Lewis index (TLI) = 0.915, comparative fit index (CFI) = 0.923, root-mean-square error of approximation (RMSEA) = 0.076, suggesting that the measures possessed robust discriminant validity.

**Table 3:** Confirmatory factor analysis results.

**Fit indices**	**χ^2^**	**df**	**χ^2^/df**	**RMSEA**	**CFI**	**TLI**	**SRMR**
**Model A (informal voice channel design as the moderating variable)**							
Four-factor model (IC;IFC;VE;VC)	785.55	318	2.47	0.07	0.93	0.92	0.05
Three-factor model (IC+IFC;VE;VC)	1,355.86	321	4.22	0.11	0.84	0.83	0.07
Two-factor model (IC+IFC+VE;VC)	2,010.53	323	6.23	0.14	0.74	0.72	0.09
Single-factor model (IC+IFC+VE+VC)	2,609.32	324	8.05	0.16	0.65	0.62	0.11
**Model B (formal voice channel design as the moderating variable)**							
Four-factor model (IC;FC;VE;VC)	828.05	318	2.60	0.08	0.92	0.92	0.05
Three-factor model (IC+FC;VE;VC)	1,371.67	321	4.27	0.11	0.84	0.83	0.07
Two-factor model (IC+FC+VE;VC)	2,062.36	323	6.39	0.14	0.74	0.72	0.09
Single-factor model (IC+FC+VE+VC)	2,650.98	324	8.18	0.16	0.65	0.62	0.11

IC, climate for inclusion; IFC, satisfaction with informal voice channel design; FC, satisfaction with formal voice channel design; VE, voice efficacy; VC, voice behavior.

### 4.2 Hypothesis testing

#### 4.2.1 Main and mediating effects testing

We used hierarchical regression analysis (see Table 4) to test the hypotheses. In Model 1 to 3, the dependent variables were voice efficacy, while those of Model 4 to 6 were voice behavior. The results showed that climate for inclusion had a significant positive influence on voice behavior (Model 4, β = 0.40, *p* < 0.001), supporting the main effect proposed in Hypothesis 1. Climate for inclusion significantly enhanced voice efficacy (Model 1, β = 0.85, *p* < 0.001), supporting Hypothesis 2. Voice efficacy positively influenced voice behavior (Model 5, β = 0.34, *p* < 0.001), supporting Hypothesis 3.

**Table 4:** Results of regression analysis and the mediating effect.

**Variable**	**VE**			**VC**		
	**Model 1**	**Model 2**	**Model 3**	**Model 4**	**Model 5**	**Model 6**
Gender	−0.17	−0.17	−0.16	−0.07	−0.04	−0.04
Age	0.01	0.01	0.01	−0.01	−0.01	−0.02^*^
Level of education	−0.17	−0.16	−0.16	−0.11	−0.09	−0.07
Working years	−0.01	−0.01	−0.01	0.01	0.01	0.01
IC	0.85^***^	0.64^***^	0.83^***^	0.40^***^		0.22^***^
VE					0.34^***^	0.21^***^
IFC		0.33^***^				
FC			0.05			
IC × IFC		0.11^*^				
IC × FC			0.14^**^			
R^2^	0.52	0.56	0.53	0.32	0.34	0.38
ΔR^2^	0.52	0.04	0.01	0.32	0.34	0.06
F	58.45^***^	48.75^***^	43.44^***^	26.25^***^	28.37^***^	28.48^***^
Indirect effects via VE	Effect		BootSE	BootLLCI		BootULCI
VE	0.180		0.036	0.109		0.251

*N* = 281.

IC, climate for inclusion; IFC, satisfaction with informal voice channel design; FC, satisfaction with formal voice channel design; VE, voice efficacy; VC, voice behavior; LLCI, Lower limit confidence interval; ULCI, Upper limit confidence interval.

^*^p < 0.05.

^**^p < 0.01.

^***^p < 0.001.

According to the method of testing the mediating effect proposed by Baron and Kenny (1986), comparing Model 4 and 6, the positive relationship between climate for inclusion and voice behavior remained significant after simultaneously including climate for inclusion and voice efficacy, and the regression coefficient increased from 0.32 to 0.38. This indicated that voice efficacy partially mediated the main effect. To validate the accuracy of the conclusions, we used a PROCESS macro (specifically Model 7) developed by Hayes (2013) to perform a bootstrapping analysis with 5,000 resamples and 95% confidence intervals. The results showed that the mediating effect of voice efficacy between climate for inclusion and voice behavior was 0.18 (*p* < 0.001), 95% CI (LLCI = 0.109, ULCI = 0.251), suggesting that the mediating effect of voice efficacy was statistically significant, which supported Hypothesis 4.

#### 4.2.2 Moderating effects testing

To avoid multicollinearity, we conducted decentralized processing on both the independent variable and the moderating variable to test the moderating effect of satisfaction with voice channel design. The results showed that the interaction term of climate for inclusion and satisfaction with informal voice channel design significantly and positively predicted voice efficacy (Model 2, β = 0.11, *p* < 0.05). The interaction term of climate for inclusion and satisfaction with formal voice channel design significantly and positively predicted voice efficacy (Model 3, β = 0.14, *p* < 0.01). This supported Hypothesis 5.

**Figure 2:**
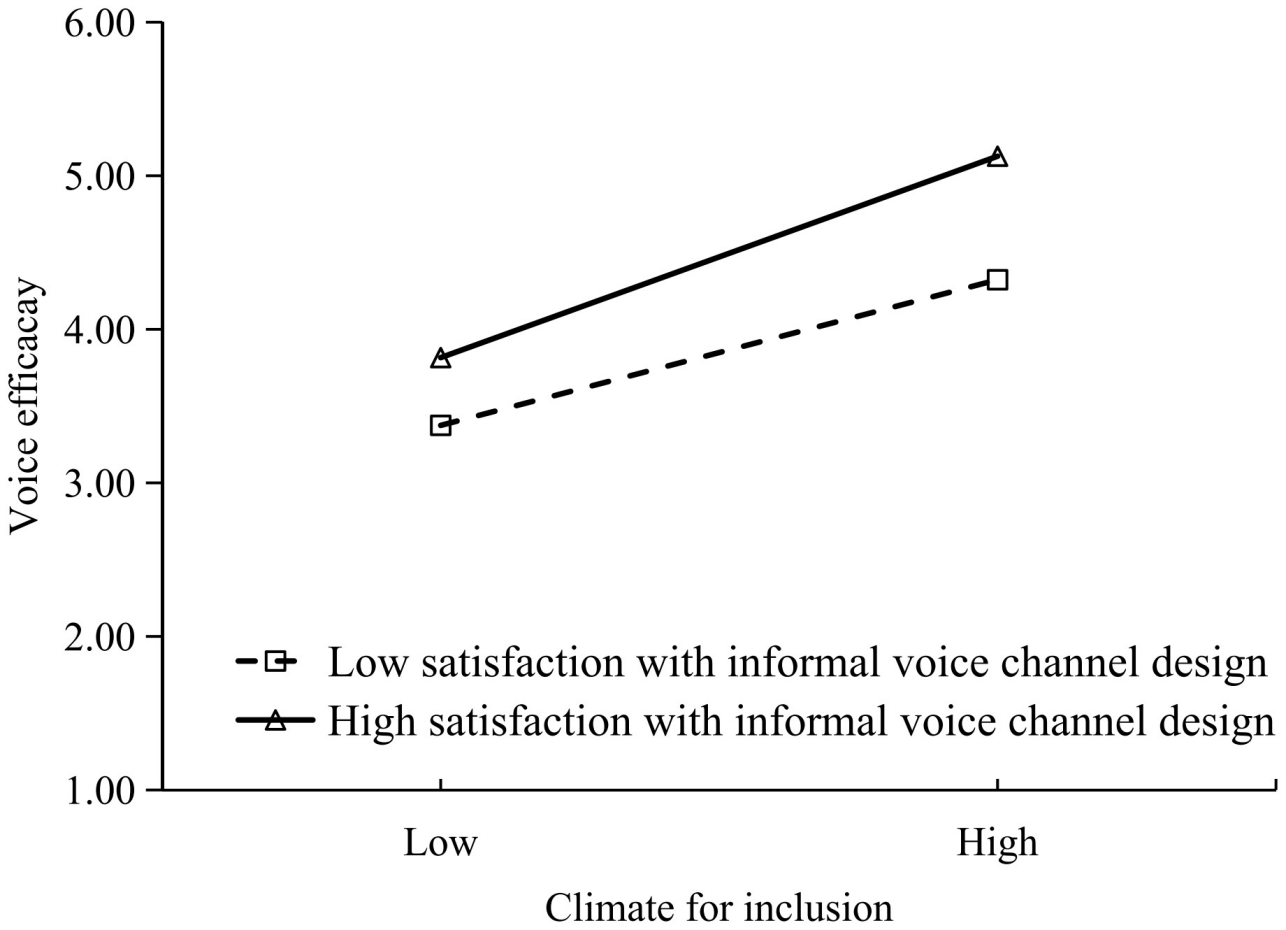
Moderating effect of satisfaction with informal channel design.

**Figure 3:**
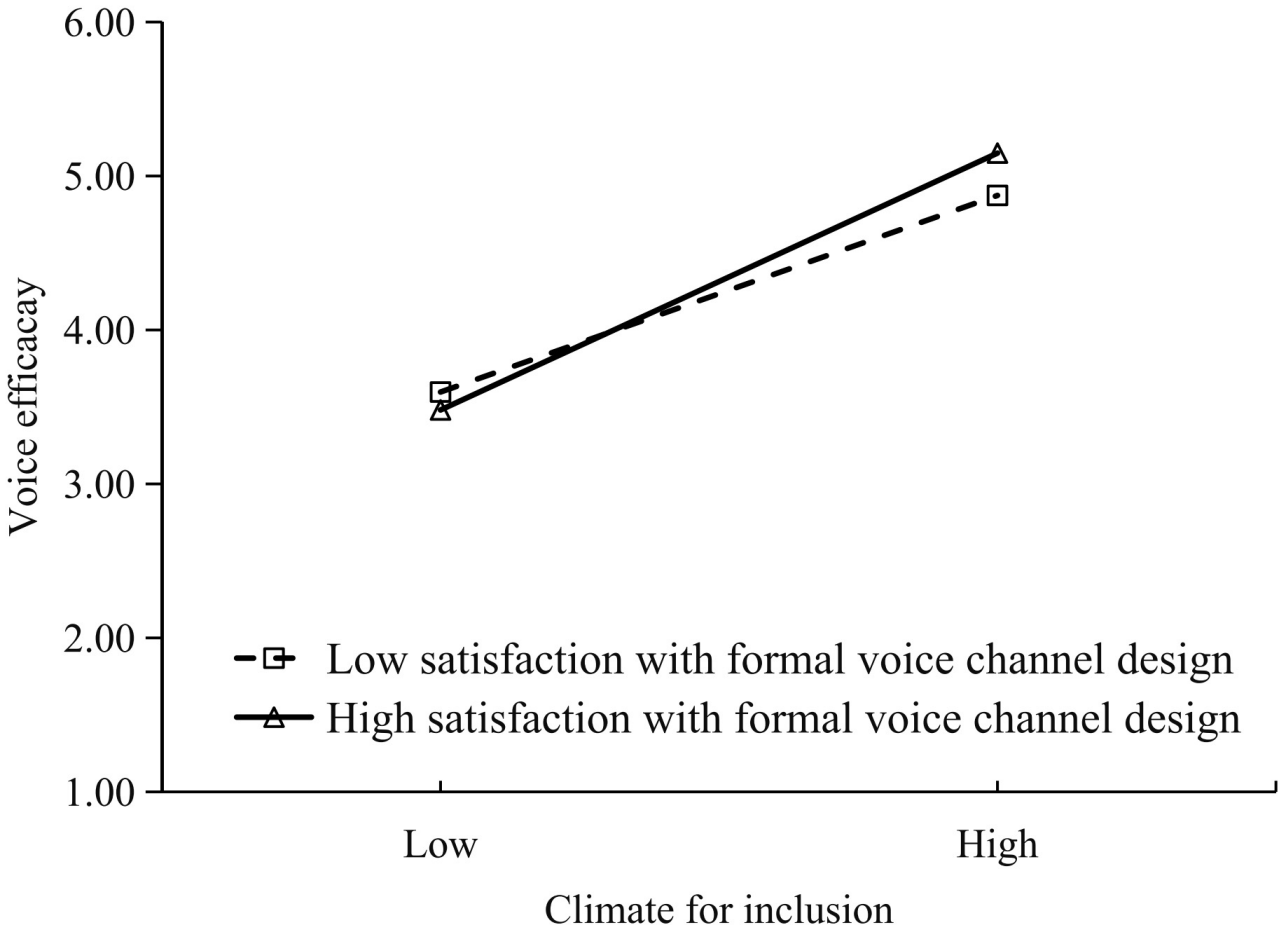
Moderating effect of satisfaction with formal channel design.

The moderating effects of satisfaction with informal voice channel design and satisfaction with formal voice channel design were respectively illustrated in Figures 2, 3 for both high (*M* + 1 *SD*) and low (*M* – 1 *SD*) levels. Regardless of whether the voice channel was informal or formal, a simple slope analysis revealed that satisfaction with voice channel design moderated the positive relationship between climate for inclusion and voice efficacy, and the relationship was stronger when employees were more satisfied with the voice channel design.

To further examine how satisfaction with voice channel design moderates the mediating effect of voice efficacy between climate for inclusion and voice behavior, Table 5 showed the index of moderated mediating effect of satisfaction with informal voice channel design was 0.02 (*p* < 0.001), 95% CI (LLCI = 0.01, ULCI = 0.06), and the index of moderated mediating effect of satisfaction with formal voice channel design was 0.03 (*p* < 0.001), 95% CI (LLCI = 0.01, ULCI = 0.06). When the satisfaction levels of informal or formal voice channel design gradually increased, the mediating role of voice efficacy gradually strengthened. It was found that the moderating effect of satisfaction with the formal voice channel design on the mediating relationship between climate for inclusion and voice behavior was slightly stronger than that of satisfaction with the informal voice channel design. This supported Hypothesis 6.

**Table 5:** Results of the moderated mediation effect analysis.

**IC**	**Effect**	**SE**	***t***	***p***	**LLCI**	**ULCI**
Direct effect	0.22	0.05	4.41	0.00	0.12	0.32
Conditional indirect effects	IFC		Effect	BootSE	BootLLCI	BootULCI
VE1	MEAN-SD		0.12	0.03	0.06	0.17
VE2	MEAN		0.14	0.03	0.08	0.20
VE3	MEAN+SD		0.16	0.04	0.09	0.24
			Index	BootSE	BootLLCI	BootULCI
Index of moderated mediation			0.02	0.01	0.01	0.06
Direct effect	0.22	0.05	4.41	0.00	0.12	0.32
Conditional indirect effects	FC		Effect	BootSE	BootLLCI	BootULCI
VE1	MEAN-SD		0.16	0.04	0.09	0.23
VE2	MEAN		0.17	0.04	0.10	0.25
VE3	MEAN+SD		0.20	0.04	0.11	0.29
			Index	BootSE	BootLLCI	BootULCI
Index of moderated mediation			0.03	0.02	0.01	0.06

*N* = 281.

IC, climate for inclusion; IFC, satisfaction with informal voice channel design; FC, satisfaction with formal voice channel design; VE, voice efficacy; VC, voice behavior; LLCI, Lower limit confidence interval; ULCI, Upper limit confidence interval.

## 5 Discussion

We find that a climate for inclusion has a positive impact on voice behavior and this effect is mediated by voice efficacy. Satisfaction with voice channel design also has a moderating effect on the relationship between climate for inclusion and voice efficacy and also moderates the indirect effect of voice efficacy on the relationship between climate for inclusion and voice behavior.

### 5.1 Theoretical implications

Our study contributes to a deeper understanding of the mechanism through which an organization's contextual factors promote the sustained and proactive behavior of individuals. In dynamic environments, where situational cues are subject to change, it is more likely that the work context will influence variations in the pattern and frequency of proactive behaviors (Bolino et al., 2012). Individuals' voice behavior should be considered as the result of sustained interaction between employees and management practices over time.

Our study provides empirical evidence that reveals that continuous proactive behavior is driven by the dual forces of improvements in the work environment and cognitive reinforcement. When examining the psychological mechanisms through which the organizational environment influences individual voice behavior, many scholars have argued that safety and effectiveness are central factors shaping individual voice-related decisions (Kim et al., 2025; Vu, 2024). Inclusive management practices are typically linked to psychological safety, whereas authorization-oriented management practices are more commonly associated with self-efficacy. Our research, however, indicates that inclusive management practices also enhance voice efficacy.

Our study also explored the boundary conditions under which satisfaction with voice channel design affects the sustainability of voice behavior. Previous studies have demonstrated that a supportive organizational climate, coupled with supportive supervisors, increased the likelihood that employees would voice their opinions (Guarin et al., 2025). Nevertheless, there has been insufficient investigation into which specific factors in prior voice experiences contribute to subsequent voice behavior. This study conceptualizes past voice experiences as the emotional responses and cognitive imprints that individuals form after proactively selecting the content and channels for speaking up. By examining the design of voice channels and the expansion of voicing opportunities, our study deepens the understanding of how the perception of voice practices can facilitate the continuation of individual voice behaviors.

### 5.2 Practical implications

A high-power distance cultural orientation is prevalent in China (Shao et al., 2013). Cultural differences in power distance are likely to exert a moderating effect on employee voice (Brockner et al., 2001). Given that employee voice enables less powerful organizational members to contribute their perspectives to decision-making processes, the level of power distance can influence the expression and effectiveness of such voice (Kwon et al., 2016). Several empirical studies have indicated that power distance negatively moderates the influence of leadership style (e.g., inclusive leadership; participative leadership) on employee voice behavior of public school teachers (Alazmi, 2024) and public servants (Qing and JinHua, 2023). In high-power distance cultures, where leaders' acceptance of employee voice may be constrained by hierarchical norms, employees may express voice through appropriate channels, helping supervisors feel comfortable and increasing the likelihood of voice adoption (Li et al., 2022). But the expectation of negative career consequences associated with voice expressed through specific communication channels is predictive of employee defensive silence (Sahin et al., 2021). Thus, the practical implications derived from this study are subject to the influence of cultural and contextual factors and to a certain extent can be generalized to other types of public institutions in China.

Our research suggests a number of recommendations. First, public institutions should create a favorable environment for employees to speak up and foster an inclusive climate that can stimulate proactive behavior. It is essential for public institutions to proactively address the psychological needs of diverse individuals, particularly the higher-order needs such as being respected and achieving self-worth. At the same time, decision-making process should consider both procedural fairness and interactive fairness to gather the wisdom of more employees.

Second, public institutions should foster individuals' positive voice experiences to enhance their self-efficacy in taking proactive action. It is essential to enhance the speed of response and improve the resolution efficiency for opinions and suggestions proposed by individuals. A linking mechanism should be established to enable individuals to provide recommendations to both all levels in the public institutions to stimulate their interest in offering suggestions.

Third, public institutions should design a complete set of voice channels and optimize mechanisms for democratic participation. From the perspective of formal channels, public institutions should guarantee individuals' rights to information, advice, participation, and supervision. Before making major decisions related to individual interests, a careful assessment and decision should be made based on widely collected opinions, and any possible deviations during the implementation of the decision should be promptly adjusted and corrected. From the perspective of informal channels, a positive and constructive relationship between leaders and members is essential for narrowing the distance between them. By inviting members to engage in in-depth discussions on the challenges faced during organizational development, their psychological contract with the organization is reinforced, thereby encouraging them to undertake extra-role behaviors that contribute to growth.

### 5.3 Limitations and future research directions

While this study makes some important contributions to the field, there are also some avenues for future research that could be considered. First, given that this research mainly collected data within the same time period and similar contexts, there is a certain degree of common method bias. Future studies should consider gathering longitudinal data and from varied sample sources to enhance the validity of the findings. Although common method bias may influence the observed effect sizes, the positive relationship between inclusive management practices and employee voice behavior remains consistent with the findings of existing meta-analyses (Li et al., 2024). Second, this study took the organizational practice of designing voice channels as the measure of the perception of voice practices. Although this scale has passed the reliability and validity tests, it still needs to be improved through in-depth empirical analysis to enhance its performance in terms of the accuracy and comprehensiveness of measuring the construct. Third, this study only measured the climate for inclusion from the individual perception level. Considering that an inclusive climate is an organization-level internal characteristic, future research should develop a cross-level theoretical framework that demonstrates the influence of climate for inclusion on voice behavior, based on a refined data collection strategy. Lastly, data were collected in China, and the scales used to measure satisfaction with voice channel design were developed within the Chinese cultural context, which may limit their generalizability to other cultural settings.

## 6 Conclusion

This study has built on sense-making theory to enhance understanding of the role of voice channel design in promoting employee voice efficacy, thereby fostering voice behaviors within an inclusive workplace climate. The results reveal a significant positive relationship between climate for inclusion and voice behavior, with voice efficacy partially mediating this relationship. The study also highlighted the importance of satisfaction with voice channel design as a moderator in this process, which suggests that well-designed voice channels play a crucial role in amplifying the benefits of an inclusive work climate. The results indicate that satisfaction with formal voice channels has a more pronounced moderating effect compared to satisfaction with informal voice channels. This could be attributed to the structured nature of formal channels, which may provide clearer pathways for employees to voice their opinions and receive feedback. By cultivating an inclusive climate and designing effective voice channels, organizations can enhance employees' confidence in their ability to make meaningful contributions and thus shed light on how prior voice experiences influence subsequent voice-related decisions. Future studies are encouraged to investigate other mechanisms and boundary conditions that influence the effects of voice channel design.

## Data Availability

The original contributions presented in the study are included in the article/supplementary material, further inquiries can be directed to the corresponding author.
